# 

**Published:** 1879-11

**Authors:** 


LUCIEN HOWE, M. D.

### CONTENTS:

PAGE.

ORIGINAL COMMUNICATIONS:

Gonorrhœa, by Geo. W. Stoner, M. D., Ass't Surgeon U. S. M. H. S., . . . . 133
Foreign Bodies on the Iris, by Lucien Howe, M. D., . . . . . . . . . 142

CLINICAL REPORTS:

Luxation of Femur on Dorsum Ilii—Reduction by Manipulation, reported by Wm. C. Phelps, M. D., . . - . . . . 145
Aneurism of Brachial Artery, reported by Herman Mynter, M. D., . . . . 147
"An Unrecognized Fracture of the Neck of the Femur," reported by C. C. Wyckoff, M. D., . . . . . . . . . 149
Imperforate Anus—the Rectum communicating with the Vagina, reported by Thos. Lothrop, M. D., . . . . . . 151

TRANSLATIONS:

Hysteria in Childhood, . . . . . 153
Four Cases of Nerve Stretching . . . 158
The Influence on Mortality of the Antiseptic Treatment in Wounds on the Head, translated by Herman Mynter, M. D., . 159

SELECTIONS:

Hysteria in Childhood, . . . . . . 160
A New Narcotic, . . . . . . . 162
Tr. Ferri Mur., . . . . . . . 162
Chrysophanic Acid in Skin Diseases, . . 162
Boracic Acid in Ammoniacal Cystitis . . 163
Obstacle to the Final Removal of the Canula after Tracheotomy in Children . . 163
Auscultation in Uterine Hemorrhage, . . 164

SOCIETY REPORTS:

Buffalo Medical Association; . . . - 165

EDITORIAL:

Midwives, . . . . . . . . . 169
Mortality Table, . . . . . . . 172
Buffalo Medical College, . . . . . 172

REVIEWS, . . . . . . . 173

Notice, by M. B. Folwell, M. D., . . . . 180

